# A Secretary of Public Health

**Published:** 1891-10

**Authors:** 


### A SECRETARY OF PUBLIC HEALTH.

At the recent (May, 1891,) Washington meeting of the American Medical Association, it will be remembered, a committee was appointed to inquire into the expedience of asking Congress to create a Bureau of Public Health: that this committee made a very full report (published in full in the JOURNAL in July) in which was set forth at great length, and most eloquently, the many reasons—looked at from a sanitary standpoint, and in the interest of preventive medicine—why such bureau should be created. This report has been submitted to the members of a national committee consisting of a distinguished representative from each State (from Texas, Dr. R. M. Swearingen is the representative), who are asked to criticise it, to give their approval, or suggest changes, etc. When the opinions of these gentlemen shall have been collated, an amended report will be formulated. This will then be sent to every reputable physician in the United States for his signature. This monster document will then be submitted to Congress, accompanied by a memorial asking that the voice of the profession be heard, and their request for recognition as an element in the law making power, be granted.

This is a most gratifying movement. We are satisfied that when Congress realizes the necessity, therein so strongly set forth, they will accede to the request, and give the medical profession representation in the Cabinet—a Minister of Public Health.

When it is reflected that two hundred years ago the death rate in Great Britain was eighty per thousand; and that in consequence of sanitary reforms it has been reduced to less than 27, it requires no argument to prove the value of sanitation. That laws are required to enforce it no one doubts: but Congressmen know nothing either of sanitary science or of the necessity of reforms, and they do not deign to consult the medical men—there is no one, in fact, who is authorized to speak. How, then, can they create laws intelligently that will correct sanitary evils? In the report, many of these evils—whence arise influences very destructive of life—are set forth. We will instance only one here; that will answer the purpose of illustration:—the pollution of streams. As the matter stands now, what is to prevent any man who chooses, from establishing a slaughter pen on the bank of any stream, no matter if this stream be the source of the water supply for whole communities? or from setting his privy on the bank of any spring, run or creek, provided he owns the land? The people do not realize the danger which results from such as this: that the water is constantly charged with the germs of typhoid fever, diphtheria, etc., and when one of these deadly diseases breaks out in a rural district, the panic stricken people wonder where it came from.

The Michigan Board of health, some years ago, through their intelligent secretary, reported the occurrence of an epidemic of typhoid fever in that State, which, upon investigation, was found to have originated in the pollution of a small river near its source. A person residing on the bank of this stream was attacked with fever; the vessels containing his excreta were emptied in the back yard on a snow bank; the snow melted, and the matter was carried into the stream. The fever made its appearance at several towns in succession, and the disease prevailed as a fatal epidemic. Doubtless, the family knew no better; but had there been a penalty, or had the family physician been a sanitarian, this epidemic would not have occurred. So, with many things. We need sanitary laws, and none but the enlightened sanitarians can dictate them.

